# Supplementary material for: MetaRibo-Seq measures translation in microbiomes
Source: Nat Commun. 2020 Jun 29;11:3268. doi: 10.1038/s41467-020-17081-z (PMC7324362; doi:10.1038/s41467-020-17081-z)
Supplement: Supplementary file 10 — Supplementary Data 7 [file 41467_2020_17081_MOESM10_ESM.zip › File2/Confidence_High_Taxonomy/241218_out.krona.html]

Javascript must be enabled to view this page.

members
magnitude
magnitudeUnassigned
count
unassigned
taxon
rank

241218\_out

4

superkingdom
4
2

phylum
4
1239

4
1263026
species

SRS011239\_contig\_number\_1286SRS013476\_contig\_number\_contig-100\_343.229522SRS016203\_contig\_number\_12369SRS078419\_contig\_number\_36727
